# Invasive Wild Pigs as Primary Nest Predators for Wild Turkeys

**DOI:** 10.1038/s41598-020-59543-w

**Published:** 2020-02-14

**Authors:** Heather N. Sanders, David G. Hewitt, Humberto L. Perotto-Baldivieso, Kurt C. VerCauteren, Nathan P. Snow

**Affiliations:** 1 Caesar Kleberg Wildlife Research Institute, Texas A&M University Kingsville, 700 University Boulevard, Kingsville, TX 78363 USA; 2 USDA/APHIS/Wildlife Services, National Wildlife Research Center, 4101 LaPorte Avenue, Fort Collins, CO 80521 USA

**Keywords:** Invasive species, Behavioural ecology

## Abstract

Depredation of wild turkey (*Meleagris gallopavo*) nests is a leading cause of reduced recruitment for the recovering and iconic game species. Invasive wild pigs (*Sus scrofa*) are known to depredate nests, and have been expanding throughout the distributed range of wild turkeys in North America. We sought to gain better insight on the magnitude of wild pigs depredating wild turkey nests. We constructed simulated wild turkey nests throughout the home ranges of 20 GPS-collared wild pigs to evaluate nest depredation relative to three periods within the nesting season (i.e., early, peak, and late) and two nest densities (moderate = 12.5-25 nests/km^2^, high = 25-50 nests/km^2^) in south-central Texas, USA during March–June 2016. Overall, the estimated probability of nest depredation by wild pigs was 0.3, equivalent to native species of nest predators in the study area (e.g., gray fox [*Urocyon cinereoargenteus*], raccoon [*Procyon lotor*], and coyote [*Canis latrans*]). Female wild pigs exhibited a constant rate of depredation regardless of nesting period or density of nests. However, male wild pigs increased their rate of depredation in areas with higher nest densities. Management efforts should remove wild pigs to reduce nest failure in wild turkey populations especially where recruitment is low.

## Introduction

Generalist invasive species negatively impact native species by both consuming and competing with the native species for resources^1,2^. In particular, the negative impact of invasive wild pigs (*Sus scrofa)*; also termed feral swine, feral hogs, or wild boars^3^; on native ecosystems has become an increasing concern as populations of wild pigs continue to expand^4^. Wild pigs were first introduced to North America by European colonists in the early 1500s^5^. Over the past 3 decades wild pigs have rapidly expanded their range from 18 to 35 of the 50 United States^6^, because of their generalist nature and continued translocation by humans^7–10^. In their expanded range, wild pigs have the potential to triple their population every 5 years in the absence of control efforts^10^, leading to detrimental effects on native species^4^. In particular, wild pigs alter habitat and compete with and prey upon native species^11–14^.

Wild pigs have been implicated as nest predators for ground-nesting birds and reptiles^15–18^. Nest failure is the most substantial limitation on population growth of ground-nesting birds, and has been predominantly attributed to nest depredation^19–21^. The impacts of specific nest predators, such as wild pigs, on the reproductive success of a ground-nesting bird can depend on predator behavior. Reports suggest that wild pigs depredate nests opportunistically^22,23^, or contrarily seek high concentrations of nests^15^, although additional reports are variable^4,18,24^. The inconsistency in findings demonstrate that the mechanisms of nest depredation by wild pigs are not well understood.

Wild turkeys (*Meleagris gallopavo*) are an iconic game species in the United States that were brought to near extinction from overexploitation during the 19^th^ century^25,26^. Intensive recovery efforts have since ensued, and in 2003 the economic impact of the spring wild turkey season was valued at $1.8 billion USD nationally^27^. With the expansion of wild pigs throughout the US, many populations of wild turkey are facing a new challenge from nest depredation e.g.^18,24^. Wild turkeys exhibit spatially-aggregated nesting behavior by selection of specific habitat structure^28^. This aggregation makes wild turkey nests particularly vulnerable to predators that actively seek nests in habitat where nesting is most likely to occur^29,30^. For this reason, it has been suggested that removing wild pigs may increase wild turkey recruitment^31^.

Simulated nests have been widely used to assess different aspects of nest depredation. However, this practice has been criticized for potential to have different depredation rates and attract different predators than natural nests^24,32,33^. While traditional transect methods for simulated nest studies may result in inflated depredation rates, simulated nest studies which place nests randomly within turkey nesting habitat have shown depredation rates similar to natural turkey nests^34^. Additionally, simulated nest studies allow for assessment of the influence of the density of nests on nest depredation^35^.

We used simulated wild turkey nests to evaluate the magnitude of nest predation by wild pigs relative to native nest predators throughout the nesting season in 2016. We also simulated differing densities of nests to evaluate for changes in the frequency of nest predation. Our first objective was compare the frequency of nest consumption by wild pigs to that of native nest predators. Secondly, we assessed the role of seasonality and density of nests in the depredation of nests by wild pigs. Finally, we evaluated differences in consumption of nests by sex and age of wild pigs. Our results will inform efforts to mitigate the impacts of wild pigs on wild turkeys.

## Methods

### Study area

Our study area was located in south-central Texas (Bexar County), USA (Latitude 29.622 N, Longitude 98.572 W). Specifically, we worked on Camp Bullis Military Reserve, an 11,000 ha military training base that is bordered by both urban and rural ecosystems. Topography on Camp Bullis is typical of the Edwards Plateau: rolling hills with limestone outcrops and rocky soils^36^. Vegetation is an oak woodland and grassland matrix^37,38^. Streams and pools are available seasonally during periods of high precipitation. Camp Bullis contains populations of both wild pigs and Rio Grande wild turkey (*M. gallopavo* intermedia), as well as potential nest predators including raccoons (*Procyon lotor*), gray foxes (*Urocyon cinereoargenteus*), coyotes (*Canis latrans*), bobcats (*Lynx rufus*), opossums (*Didelphis virginiana*), striped skunks (*Mephitis mephitis*), armadillos (*Dasypus novemcinctus*), corvids, and snakes^23,28^. In 2006, a suspected release or immigration from surrounding properties of wild pigs occurred on Camp Bullis, resulting in abundant wild pigs throughout the study area (M. L. Cooksey, U.S. Army Environmental Command, personal communication). In 2017, the density of wild pigs was reported to average 3.5 wild pigs per km^2^ ^39^.

### Capture and monitoring of wild pigs

Methods for this study were similarly reported for a simultaneous study^23^. We captured wild pigs using corral and box traps baited with whole-kernel corn between 15 January and 15 March 2016. We immobilized adult wild pigs (estimated to be >45 kg) using an intramuscular injection of 3.3 mg/kg Telazol® (Zoetis, Parsippany, New Jersey, USA) and 1.5 mg/kg xylazine hydrochloride (Wildlife Pharmaceuticals, Inc., Windsor, Colorado, USA^40^). We applied uniquely identifiable ear tags (Allflex® A Cattle Tags, Allflex USA Inc., Dallas, Texas, USA) and Global Positioning System (GPS) satellite-transmitting collars (VERTEX PLUS-2 Collar, VECTRONIC Aerospace GmbH, Berlin, Germany) to the immobilized wild pigs. After handling was complete, we intramuscularly injected 0.2 mg/kg yohimbine hydrochloride (Wildlife Pharmaceuticals, Inc., Windsor, Colorado, USA) as a reversal agent^40^. To ensure spatial coverage of GPS-collared wild pigs across Camp Bullis, we marked ≤2 wild pigs from any social group captured, and we moved traps regularly into locations where wild pigs had not yet been marked. All capture and handling procedures were approved by the Texas A&M University-Kingsville’s Institutional Animal Care and Use Committee (2015-08-20), and all methods were performed in accordance with guidelines outlined in that protocol.

We programmed the GPS collars to record and store locations at 15-minute intervals throughout the study period. Every sixth location was transmitted via Iridium satellite (Iridium Communications, Inc. McLean, Virginia). A drop-off mechanism on each collar was programmed to automatically disconnect the collars on 15 August 2016, after which the full dataset was retrieved from onboard storage. Overall, we attached GPS collars to 35 wild pigs (17 males and 18 females); seven used inaccessible areas (i.e., adjacent private lands), four collars slipped off, one collar failed, and one collared wild pig was killed by an adjacent landowner during the study. Movements of two collared male wild pigs were expansive, covering most of the study area, and could not be targeted for simulated nest deployment. Therefore, we ultimately considered 20 wild pigs (13 males and 7 females) as our primary study animals.

We calculated real-time home ranges for each wild pig every 14 days using transmitted locations from the previous 28 days to inform our placement of simulated nests. Specifically, we calculated the 95% minimum convex polygon home ranges using package adehabitatHR^41^ in Program R (v3.3.1, www.r-project.org, The R Foundation, Vienna, Austria^42^;). We used minimum convex polygons because this method conservatively estimates a maximum extent of space-use for each animal^43^, ensuring our placement of simulated nests would target the entirety of areas used by collared wild pigs.

### Nesting habitat suitability model

We built a habitat suitability model to guide our placement of nests within the home ranges of wild pigs using published metrics of nest site selection in wild turkeys^44,45^. Specifically, we used ERDAS Imagine® (Hexagon Geospatial, Norcross, Georgia) to classify aerial imagery from the 2014 National Agriculture Imagery Program with 1 m resolution (USDA-FSA-APFO Aerial Photography Field Office, Salt Lake City, Utah) from 4 images into woody and herbaceous cover. We conducted an accuracy assessment using 1,200 ground control points and determined the overall accuracy was 89%. We imported classified images into FRAGSTATS v4.2^46^; and used moving windows analyses with a 100-m radius for woody cover on the following metrics: percent land cover, edge density, patch density, and largest patch index. We used ArcGIS (v10.3.1, Environmental Systems Research Institute, Inc., Redlands, CA, USA) to combine the resulting landscape metrics layers and reclassify them into nesting and non-nesting habitat. A location was considered suitable for nesting if the surrounding 100 m had woody cover of 10 to 70%, edge density of >75 m/ha, patch density of >50 patches/100 ha, and a largest patch index of <50%^44,45^.

### Placement, construction, and monitoring of simulated nests

We divided the 20 collared wild pigs into nest density treatments (9 = moderate, and 11 = high) based on non-overlapping space-use so that the density treatments did not overlap. We generated random points using ArcGIS to select the locations of nests within each home range. We randomly placed simulated nests throughout areas identified as nesting habitat within the home ranges of collared wild pigs. We modified selection of the nest sites on the ground to be ≤50 m from each generated random point and best represent true nesting habitat by selecting for small patches of woody cover with dense understory^44^. We randomly assigned two treatments for density of nests to the home ranges of individual wild pigs, including: 1) moderate density with 25 nests/km^2^ nesting habitat, and 2) high density with 50 nests/km^2^ nesting habitat). We based our moderate density treatment on an estimated density of turkeys in oak savanna habitat in south Texas of 13 birds/km^2^ with an estimated sex ratio of 45% female^47,48^, and an estimate of 97% initiation of nests by hens^49^. We corrected the estimated nests/km^2^ by the proportion of the study area that was designated as nesting habitat according to our habitat suitability model. We doubled the moderate density value to determine our high density treatment and to evaluate the response of wild pigs to extremely high concentrations of wild turkey nests.

**Figure 1:**
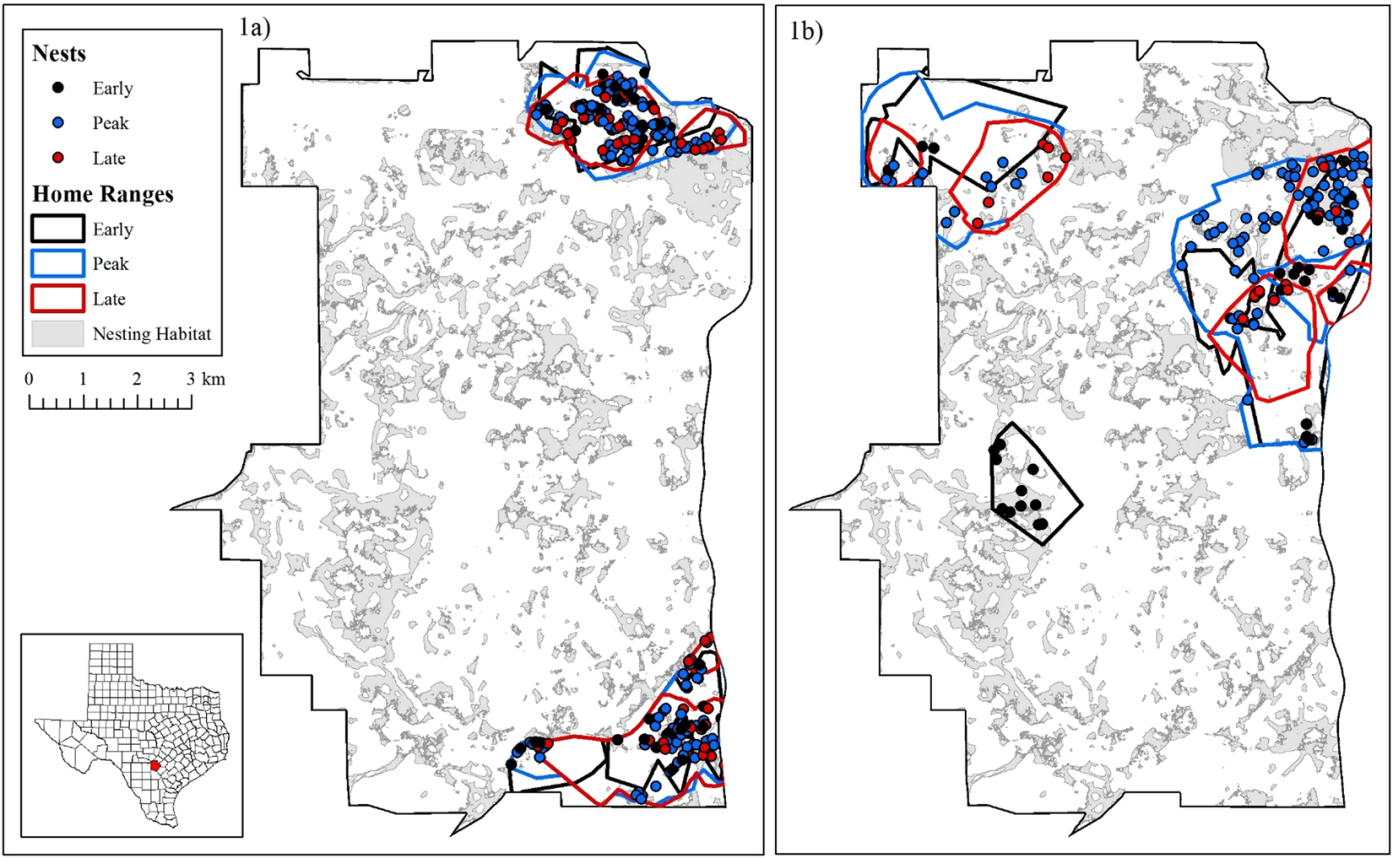
Treatment areas and subsequent distributions of nests within the study area in Bexar County, Texas, USA during the early, peak, and late nesting periods for (**a**) the high density, and (**b**) the moderate density treatment areas. Treatment areas were delineated by combining overlapping home ranges (95% minimum convex polygon) of wild pigs to create contiguous areas for each nesting period.

We utilized random block design to mimic natural nesting chronology of wild turkeys and assess the role of timing within nesting period while maintaining our experimental density treatments. Specifically, we split the nesting season into three 28-day nesting periods including: (1) early period from 28 March to 24 April, (2) peak period from 25 April to 22 May, and (3) late period from 23 May to 20 June. Therefore, the simulated nesting density for the moderate density treatment varied from: early = 12.5 nests/km^2^, peak = 25 nests/km^2^, and late = 12.5 nests/km^2^. Whereas, the high density treatment varied from: early = 25 nests/km^2^, peak = 50 nests/km^2^, and late = 25 nests/km^2^. Overall, we constructed 384 simulated turkey nests between 28 March and 20 June 2016 within the home ranges of collared wild pigs, including 137 nests in the moderate and 247 in the high density treatments (Fig. 1).

We constructed simulated nests using unwashed domestic chicken (*Gallus domesticus*) eggs, which are similar in both size and shape to wild turkey eggs^50^. We placed 10 eggs in a shallow nest bowl lined with leaves. We made no attempt to obstruct the nest with leaf litter. To minimize the addition of human scent, we wore clean rubber soled boots and latex gloves at all nest sites and while handling equipment. We monitored nests for evidence of depredation every seven days for 28 days. If a nest survived 28 days, we removed the nest and placed a new nest at a new random location to maintain density. Similarly, if a nest was depredated, we placed a new nest in a new random location within the same home range to maintain consistent nest density treatments within the home ranges throughout the study.

We randomly selected and monitored a subset of 50 nests using motion detecting cameras (Reconyx PC900 Hyperfire™ Professional Covert IR Cameras, RECONYX, Inc., Holmen, Wisconsin) placed approximately 25 cm above the nest and 2 m away to identify the predators of nests. For nests with >1 species of predator, we identified the primary predator based on which species spent the most time depredating. We also recorded the number of encounters for each species of predator, which we defined as any time a predator approached within ≤1 m of the nest bowl. Finally, we determined the sex and age class (adult or juvenile) of each observed wild pig by examining their secondary sex characteristics, where adults had enlarged, protruding testes or nipples suggesting sexual maturity. We did not identify sex of juvenile wild pigs. We characterized foraging by wild pigs by sex and age class (adult or juvenile) observed participating in depredation.

### Data analysis

We used binomial generalized linear models with logit links (glm) in Program R to evaluate whether our study methods seemed to influence our results. First, there are conflicting reports that cameras on nests may repel predators^51,52^, or possibly alert predators to the location of monitored nests and inflate predation rates^24^. Therefore, we analyzed the effect of camera monitoring on the probability of depredation to determine if the sample of nests monitored with cameras was representative of those without cameras. Second, we also recognized that eggs may have become more odorous over time, which may increase the rate of depredations. Therefore, we analyzed whether the number of days a nest had been active influenced the probability it was depredated by a wild pig.

We also compared the proportions of nests depredated by each species of predator using Fisher’s exact test. Then, we used binomial glms to evaluate for influences from the nesting period and density of nests, and the interaction of period × density, on the probability of depredation by each species of predator. We also predicted the probability that a nest would be depredated for each nesting period and nest density treatment for all species of nest predators pooled, and for each species of nest predator individually. Additionally, we predicted the probability a nest would be depredated by male and female wild pigs.

We used a binomial hypothesis test to assess the hypothesis that wild pigs will consume a nest once they discover it (i.e., observed on camera at the nest). Specifically, we compared the number of times wild pigs depredated a nest to the number of times wild pigs approached a nest ≤ 1 m but did not depredate the nest. We compared the ratio of foraging events to the number of times a nest was discovered among predator species using Fisher’s exact test. We used the level of α = 0.05 to represent statistical and biological influences for all tests.

## Results

We observed that 300 of 384 simulated nests (78.1%) were partially or wholly depredated. Nests with cameras did not have a different depredation rate (75.0%) than those without (78.3%; F_1, 382_ = 0.285, *P* = 0.594). We also found no evidence that increasing odors from the eggs increased predation rates through time. The estimated odds that a nest would be depredated by wild pigs decreased by 6.8% for every day a nest was active (F_1, 1378_ = 10.19, *P* = 0.001).

From the 52 nests monitored with cameras, we identified 6 wildlife species that depredated nests, including wild pigs, gray foxes, raccoons, coyotes, striped skunks, and a nine-banded armadillo. Wild pigs depredated 15 of the 52 (29%) nests. Gray foxes (21%), raccoons (19%), and coyotes (17%) were also frequent nest predators (Table 1). In all cases, the primary nest predators were also the first nest predators to visit the nests. We also observed nest depredation by an unidentified snake species during manual nest checks but did not observe via camera. We only recorded 1 observation of depredation by an armadillo, therefore we excluded armadillos from further analysis. In seven instances of nest depredations we could not identify the predator.

**Table 1:** Number (and percent) of nests where depredation was recorded and for which that species was the primary nest predator observed in Bexar County, Texas during 2016.

Predator Species	Depredation	Primary Depredation
Wild Pig	15 (29%)	12 (23%)
Gray Fox	11 (21%)	7 (13%)
Coyote	9 (17%)	6 (12%)
Raccoon	10 (19%)	5 (10%)
Striped Skunk	4 (8%)	2 (4%)
Armadillo	1 (2%)	0 (0%)
Unidentified	—	7 (13%)
Total	—	39 (75%)

**Figure 2:**
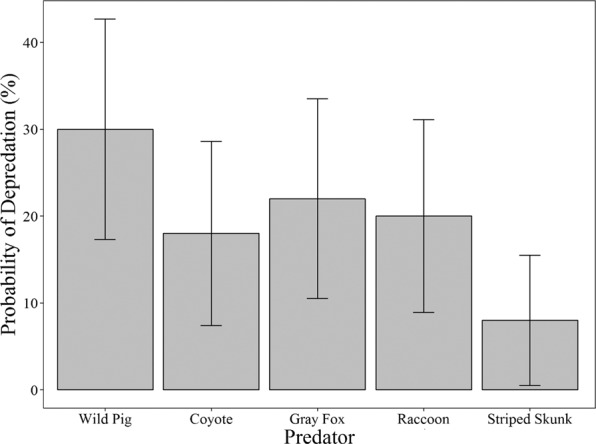
Predicted probability that a nest will be depredated by each species of nest predator in both primary and secondary depredation events with 95% confidence intervals for the 2016 wild turkey nesting period in Bexar County, Texas.

**Figure 3:**
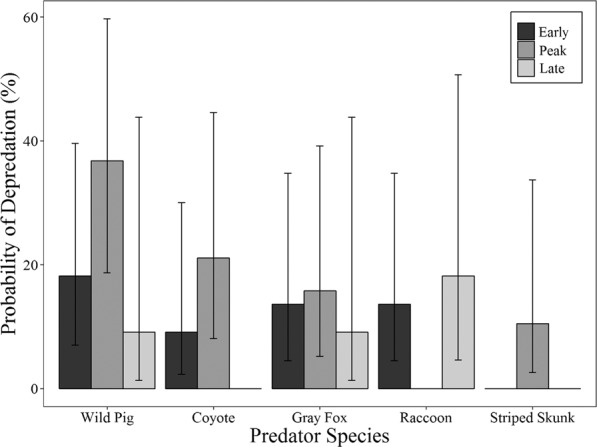
Predicted probability that a nest will be depredated by each species of nest predator during each nesting period with 95% confidence intervals for the 2016 wild turkey nesting period in Bexar County, Texas.

**Figure 4:**
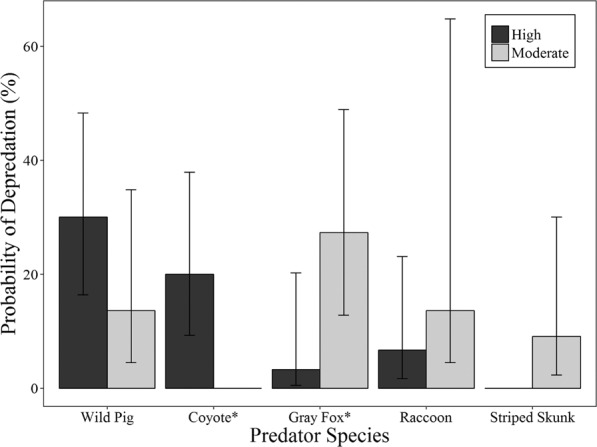
Predicted probability that a nest will be depredated by each predator species for each density treatment with 95% confidence intervals for the 2016 nesting period in Bexar County, Texas. For each species, an asterisk indicates a significant (*P* < 0.05) difference between nest densities.

We found that no species of nest predator depredated a higher proportion of nests than another species (two-sided *P = *0.079). Similarly, the 95% CIs on the predicted probabilities of depredation overlapped for wild pigs, coyotes, gray foxes, and raccoons (Fig. 2). The interaction of period × density was not significant for any species of nest predator, therefore we excluded the interaction from all models. For all nests, the probability of nest depredation varied by period with the highest probability during peak nesting (F_2, 381_ = 29.88, *P < *0.001; Table 2), but did not vary by density treatment (F_1, 382_ = 1.437, *P = *0.231). The probability that a nest was depredated by a wild pig did not differ by nesting period (F_2, 49_ = 1.788, *P = *0.178; Fig. 3) or by density treatment (F_1, 50_ = 1.911, *P = *0.173; Fig. 4).

**Table 2:** Number of nests depredated and predicted probability a nest would be depredated by any predator species, all invasive wild pigs, male wild pigs, and female wild pigs with the lower confidence level (LCL) and upper confidence level (UCL) for a 95% confidence interval for each period and density treatment in Bexar County, Texas during 2016.

Treatment	Predator	No. nests depredated	Probability of Nest Depredation	LCL	UCL
**Period**					
Early	All Species	61	0.592*	0.495	0.683
	Wild Pigs	4	0.182	0.07	0.396
	Male	2	0.091	0.023	0.300
	Female	2	0.091	0.023	0.300
Peak	All Species	198	0.912*	0.867	0.943
	Wild Pigs	7	0.368	0.187	0.597
	Male	4	0.21	0.081	0.446
	Female	3	0.158	0.052	0.392
Late	All Species	40	0.625*	0.501	0.734
	Wild Pigs	1	0.091	0.013	0.483
	Male	0	0	0	1.000
	Female	1	0.091	0.013	0.439
**Density**					
High	All Species	197	0.792	0.743	0.843
	Wild Pigs	9	0.3	0.164	0.483
	Male	6	0.200*	0.093	0.379
	Female	3	0.1	0.033	0.268
Moderate	All Species	102	0.745	0.665	0.811
	Wild Pigs	3	0.136	0.045	0.348
	Male	0	0.000*	0	1.000
	Female	3	0.136	0.045	0.348

Probabilities include only initial predation. For each predator group, statistically distinct probabilities within a treatment are marked with an asterisk.

Wild pigs depredated nests during 58.2% of observed encounters with nests. This was not significantly different from the proportion depredated at discovery by raccoons, gray foxes, or coyotes (two tailed *P = *0.071). Probability of depredation given that a nest had been discovered did not vary by sex (F_2, 149_ = 0.068, *P = *0.935) or age class (F_2, 149_ = 0.302, *P = *0.739) of wild pigs. The probability that a nest would be depredated by a male wild pig was substantially greater in the areas with a high versus moderate densities of nests (F_1, 50_ = 5.288, *P = *0.026), but did not differ among nesting periods (F_2, 49_ = 1.633, *P = *0.206; Table 2). The probability that a nest would be depredated by a female wild pig did not differ by density or nesting period (F_2, 49_ = 0.252, *P = *0.778; F_1, 50_ = 0.159, *P = *0.692; Table 2).

## Discussion

Our finding that nearly 80% of simulated wild turkey nests being depredated is concerning for wild turkey populations in areas with dense populations of wild pigs. Wild pigs were responsible for depredating as many nests as the most frequently observed native nest-predator, depredating nearly 30% of nests. These results are contrary to previous reports that wild pigs are minor nest predators relative to native species e.g.^28,53^, which leads us to re-pose the question of whether depredation by wild pigs is additive to that observed by native predators. In the absence of wild pigs, depredation rates of non-artificial wild turkey eggs ranged widely from 18–65%^54–56^, but were always lower than the 80% we observed with artificial eggs and a dense population of wild pigs present. For other ground nesting species (e.g., sea turtles), the addition of wild pigs with native predators did appear to produce additive nest predations^57,58^. Wild pigs were also frequently the first species to visit the nests in our study, which could likely lead to nest abandonment even if not all eggs were destroyed. Therefore, we suggest that wild pigs may be causing additive nest failures for wild turkeys and more research is needed to test this hypothesis.

Wild turkey nests had substantially higher probabilities of predation by male wild pigs in areas with higher nest densities. This could be indicative of males showing an adaptive response to the pulsed resources of nests, similar to nest depredation behaviors reported in other studies e.g.^15,18,57^. Wild pigs are known to exploit seasonal resources, such as ground nests, as those resources become available^59,60^. However, male wild pigs also moved farther distances and had larger home ranges than females in this study area^61,62^, suggesting that males could have discovered more nests randomly during their increased movements.

Female wild pigs did not appear to increase predation pressure on nests seasonally or in response to increased nest density. Our attempt to mimic natural wild turkey nesting behaviors, by dynamically adjusting nest densities throughout nesting periods, may have reduced our ability to detect responses to either variable. However, our results corroborate similar findings that wild pigs did not actively seek nests regardless of nest density or period^23^, therefore we do not expect this was the case. More research on the behaviors of wild pigs is needed to conclude whether males exhibited an adaptive response to wild turkey nests, and females did not.

Our results paired with previous studies establish that wild pigs fulfill three detrimental roles for wild turkeys (i.e., consume nests, compete for resources, and alter habitat), and have the potential to reduce turkey populations^11,13,63,64^. Wild turkeys are not the only ground-nesting species susceptible to nest depredation by wild pigs e.g.^15,18,57^, particularly as the range of wild pigs continues to expand throughout the US and worldwide. For example, Snow, *et al*.^9^ predicts that wild pigs have a high propensity to invade portions of the nesting range of a number of sensitive species including piping plovers (*Charadrius melodus*), greater sage-grouse (*Centrocercus urophasianus*), and northern pintails (*Anas acuta*)^65–67^. Additionally, long-term studies of various North American turtle species indicate that additive mortality of as little as 3% may result in population decline, suggesting turtles may be highly sensitive to depredation by wild pigs^68,69^. Halting the range expansion of wild pigs may be essential to the conservation of a variety of ground-nesting species.

While coyotes and gray foxes appeared to respond to our density treatments, this relationship is complicated by predator interactions between coyotes and gray foxes which can result in avoidance by gray foxes^70^. To our knowledge, there is no evidence that wild pigs avoid interactions with other nest predators, thus we did not expect that predator interactions impacted the observed probability a nest would be depredated by wild pigs. Contrarily, if other predators practiced avoidance in response to wild pigs, this could lower the overall depredation rate for wild turkey nests. Avoidance of wild pigs by other nest predators could also introduce bias in nest studies that only monitor nests in areas where wild pigs are known to be active. However, as wild pigs are ubiquitous throughout our study site, we believe our results accurately depict the potential impact of wild pigs on wild turkey nest success where wild pig density is high.

It is important to note that this study utilized simulated turkey nests. While simulated nest studies have shown depredation rates similar to natural turkey nests^34^, it is difficult to draw concrete conclusions regarding predation rates in the absence of the hen^32^. The presence of hens has increased predations at nests because the hen can be prey itself^54^, resulting in nest failure. In addition, the use of surrogate eggs could have led to eggs rotting over time and becoming more detectable, although we found no evidence that this increased detection by wild pigs. Contrarily, we found that probabilities of depredation decreased through time. It is unclear why this relationship existed, but we suspect it was related to some nests being placed in locations that were randomly less detectable by nest predators, despite our best attempts to place all nests equally within turkey nesting habitat. The relationship was likely not related to diminishing human disturbance through time because each nest was revisited once per week. It was also not related to changes in densities of available nests because we replaced any depredated nests with new nests, therefore keeping densities consistent. Ultimately, the use of simulated nests allowed us to evaluate the influence of treatment of nesting densities and periods, which would not have been feasible using natural nests e.g.^35^.

## Conclusions

Invasion of wild pigs into new regions may result in increased nest mortalities for wild turkeys, thus curtailing the expansion of wild pigs is highly important for protecting wild turkeys and other ground-nesting species. Efforts to protect wild turkeys by controlling wild pigs should occur immediately prior to nesting period. Determining whether nest depredation by wild pigs is additive relative to native nest predators represents an important line of future research. Further investigation of potential behavioral responses of male wild pigs to increased availability of nests are also needed to understand the impact of wild pigs on reproductive success of wild turkeys.

## Data Availability

The datasets generated during and/or analyzed during the current study are available from the corresponding author on reasonable request.
